# Pennsylvania State Veterinary Medical Association

**Published:** 1900-04

**Authors:** 


### SOCIETY PROCEEDINGS.

### PENNSYLVANIA STATE VETERINARY MEDICAL ASSOCIATION.

THE annual meeting of this Association was held at the Veterinary Department of the University of Pennsylvania, March 6 and 7, 1900. Meeting called to order at 10 A.M. by President J. C. Michener, of Colmar. The following members were present: Drs. John W. Adams, W. G. Benner, E. E. Bittles, S. P. Bishop, John L. Bradley, Francis Bridge, A. O. Cawley, M. J. Chrisman, M. J. Collins, M. E. Conard, John M. Courtright, H. B. Felton, J. T. Ferley, J. C. Foelker, S. J. J. Harger, Jacob Helmer, F. F. Hoffman, C. W. Holley, W. Horace Hoskins, J. D. Houldsworth, J. B. Irons, George B. Jobson, H. P. Keely, W. S. Kooker, L. O. Lusson, Clarence J. Marshall, J. T. McAnulty, James R. Mehaffey, W. A. Meredith, J. C. Michener, R. T. Mumma, Otto Noack, Leonard Pearson, A. W. Radley, E. M. Ranck, T. B. Rayner, James B. Rayner, John B. Raynor, N. Rectenwald, N. E. Reinhart, R. G. Rice, W. L. Rhoads, W. H. Ridge, J. W. Sallade, A. F. Schrieber, B. F. Senseman, G. K. Swank, E. E. Tower, H. W. Turner, B. M. Underhill, James A. Waugh, Charles Williams. Visitors: Drs. T. J. Kean, Philadelphia, Pa.; Elias A. Gruver, Richlandtown; C. J. Waidner, Hellertown; M. Moriarty, Gettysburg; F. F. Shue, Yorkana; L. E. Meade, Tunkhannock; N. H. Allis, Wyalusing; C. F. Keiter, Williamstown; W. H. Fry, Pine Grove Mills; W. B. Collom, Doylestown, Pa.; Ernest Spaeth, Gillette, Wyoming; H. P. Eves, Wilmington, Del.; Mr. Wm. Hammond, Glenolden, Pa.; Messrs. R. Y. Davison. J. W. Hayman, G. W. Horner, Wm. Hughes, John M. McCloskey, L. A, Nolan, O. M. Norton, E. W. Powell, B. H. Tallman, C. S. Shove, Frederick Stehle, Jr., C. R. Walter, Thomas White, students in attendance at the Veterinary Department of the University of Pennsylvania.

The minutes of the previous meeting were read and approved.

The election of officers for the ensuing year was as follows: President, S. J. J. Harger; First Vice-President, A. W. Radley; Second Vice-President, L. O. Lusson; Third Vice-President, N. Rectenwald; Recording Secretary, C. J. Marshall; Corresponding Secretary, E. M. Ranck; Treasurer, Francis Bridge; Trustees, Drs. Leonard Pearson, W. Horace Hoskins, George B. Jobson, Thomas B. Rayner, and F. F. Hoffman.

The following applications for membership were received: J. P. Stover, W. H. Mattson, S. W. Matthews, J. C. Kingsland, E. A. Gruver, N. H. Allis, C. J. Waidner, E. M. Crawford, M. A. Davies, C. F. Keiter, E. S. Muir, L. A. Klein, W. B. Prothero, M. Moriarty, D. A. Gorman, J. G. Bethune, and T. J. Kean. These applications were favorably reported by the Board of Censors and accepted as members of the Society. Dr. J. C. Kingsland was accepted as a member under the following condition, that he discontinue preparing and offering for sale his proprietary medicines. The Secretary read a letter from Dr. J. P. Vasey in reference to joining the Association, his name having been proposed at the last meeting. The letter was referred to the Board of Censors, who rejected his application.

The Secretary also read a letter from Dr. Zeno S. Keil, who regretted his inability to attend the meeting on account of sickness in the family.

Delegates from other societies were called, but none responded.

The Treasurer, Dr. Francis Bridge, reported that he had carried out the suggestion, made by himself at the last meeting, of giving a bond for the safe-keeping of the funds belonging to the Society. The first interest ever realized on the funds of the Association amounted to $4.25. The treasury is in the best financial condition in its history.

*Reports of Committees.*

Dr. W. H. Ridge reported from the Committee on Legislation, that the most important thing at present was the Army Legislation Bill, and emphasized the importance of filling out the petitions sent out by Dr. R. S. Huidekoper, and returning them at once to him, so that they might be presented immediately to the Senators.

The Committee on Sanitary Science and Police made no report. Dr. Ranck called attention to the fact that the Chairman of this Committee failed to inform the other members of the importance of collecting material for a report to be made at this time. It might be well to note here that chairmen of the several committees should make a special effort to notify the other members of the importance of getting their reports ready for the meeting.

Members of the Committee on a Relief Association, appointed at the last meeting, were not present, and the President then appointed Drs. Hoskins and Rayner on this committee. Drs. Rayner and Noack were opposed to this feature. Dr. Hoskins made some very forcible remarks in favor of such body. The committee simply reported progress.

Dr. Pearson spoke, in reference to the Committee on Legislation, that the next committee should be appointed with power to act, as the Legislature would be in session before our next meeting. The motion was moved, seconded, and accepted by the Association.

At this point the meeting adjourned for lunch at the University Restaurant, which the city veterinarians had prepared as a courtesy to the members from out of town.

*Afternoon Session.*

The meeting was called to order at 2 o'clock by the President.

Dr. Felton read a paper on "Urethral Calculi in Dogs." This interesting paper was discussed by many of the members present, and interesting cases were cited by the President, Dr. J. C. Michener, Drs. Otto Noack, Ranck, and Harger.

Dr. Ranck described the bacteriology of "swine-plague and hog-cholera," and told how the preventive inoculations of hog-cholera and swine-plague were carried out by the Bureau of Animal Industry. The results of this treatment have not been encouraging.

Dr. Goentner was to have read a paper on the subject of "Osteoporosis." The doctor not being present, the subject of osteoporosis was opened for discussion. President Michener, speaking on the subject, gave as his belief that it was due to too high feeding and lack of work, and believes that we should treat all cases by discontinuing grain and turning out at pasture, if possible. Dr. Kooker also reported several interesting cases of osteoporosis. Dr. Schreiber believes they should be killed at once when they show symptoms of osteoporosis. Drs. Waugh, Mehaffey, Rogers, Eves, Lusson, and Noack all made interesting remarks on this subject. Many questions were referred to Dr. Pearson by those taking part in this discussion. Dr. Pearson, in answering these questions, said many of these points were unsettled. He, however, made reference to the interesting investigations made at the Johns Hopkins University and also by Dr. White, of the Veterinary Department, and cited a case in Honolulu, reported in the English veterinary journals, which showed that the disease is of a contagious nature, and claims that it can be cured by moving from low lands to higher altitudes. He also spoke of a number of horses sent from here to Virginia as hopeless cases, which he saw last summer, and nearly all had recovered. He advises treatment by a change of locality, if possible, and thinks we should also take the sanitary precautions suitable for combating contagious diseases. Some experiments were carried out by himself at the University two years ago, in which he injected well horses with specimens taken from a horse afflicted with this disease, and none of them took the disease.

Dr. Ranck read his paper on "Snakes, Snake-venom and Antivenine." Several of the members discussed this paper and asked questions which were answered by the essayist.

Dr. Rogers reported his experience with antipneumococcic serum. Out of nine cases treated seven recovered; 25 c.c. were given in one dose, which was not repeated.

Dr. Rectenwald reported his treatment for purpura hemorrhagica with the soluble collodiale of silver, with good results. Dr. Hoskins used the same treatment with equal success.

The appointment of librarian was discussed by the members, which resulted in the election of Dr. Hoskins.

Meeting adjourned at five o'clock.

Most of the members attended clinic the next morning before the meeting was called at 10.45. A horse with a peculiar heart-beat was exhibited and examined by most of the members present. In the absence of Dr. Robert Meade Smith, his paper on the subject of "Feeding Dairy Cattle" was read by Dr. Leonard Pearson. The paper was highly appreciated and thoroughly discussed. Dr. Jobson spoke of the importance of exercise for animals. In making a record in the production of milk and butter some animals were fed four times a day and given pepsin, one feeding being at midnight.

Dr. John W. Adams gave an interesting discussion on the subject of "The Eye," and illustrated his remarks with charts and showed how to use the ophthalmoscope, and emphasized the fact that this instrument was of little practical use to the veterinarian, and spoke of the importance of examining the eyeball from different directions, to compare the convexity and size of the eyeball; also the color of the iris should be observed as well as the pupillary margin, the black spots on the lens, etc. In examining the eye we should have the horse facing the light, with a dark background and as few objects in front of the horse as possible, as reflections from these objects tend to confuse the observer. The subject being open for discussion, many questions were asked, and some of the members reported the favorable use of Lugol's solution in the treatment of periodic ophthalmia by injecting one drachm above the orbit.

There was considerable discussion as to the use of the treatment of goitre with Lugol's solution. Dr. Waugh, of Pittsburg, claims to have had bad results from this treatment. Dr. Adams advised the use of equal parts of alcohol and iodine, which should be given in increasing doses, injected directly into the gland, then wait until the swelling subsides and repeat the treatment. The surgical removal of the gland is not advisable.

The following places were recommended for the semi-annual meeting, which is to be held on the third Tuesday in September: Erie, Allentown, Trevose, and West Chester. Erie received the largest vote, and it was decided to hold the next meeting there.

Dr. Kean read an interesting paper on the subject of "Horseshoeing," and advocated the usefulness of the rolling-motion shoe.

The annual report of the Corresponding Secretary was then read, which was as follows:

*Annual Report of the Corresponding Secretary for the Term ending March* 7, 1900.

MR. PRESIDENT AND FELLOW-MEMBERS OF THE PENNSYLVANIA STATE VETERINARY MEDICAL ASSOCIATION: In making this my final report as Corresponding Secretary of what I believe to be the most energetic and prosperous State Association in existence, I am free to say I do so with a certain feeling of regret, as my work for the past three years has brought me into close touch with every member of our Association as well as a large number of veterinarians throughout the State who are as yet not of our number, thus creating a fraternal feeling which cannot be felt or appreciated by one who has not become thoroughly interested in Association affairs. Yet I give up the arduous labors of Corresponding Secretary with a feeling of thankfulness, of sincere pride, and gratification. Thankful for the assistance of every member who has responded to my appeals for money—and I have asked for it many times—or literary contributions, for demonstrations; for we know that cash alone, though very essential, will not make an association a success when it is laboring, as we are, for the advancement of the most noble profession on earth from a sanitary, scientific point of view. Thankful that these men have responded at all times so readily, for I must admit many times the notice was very short, indeed. Thankful for the hearty support given me at all times by my brother officers. Thankful especially to our brother-members Drs. Pearson and Hoskins for the many helps they have extended to me throughout my tenure of office.

I am proud that the Association, through your missionary work (and I know full well at many times it has been laborious), has within the last three years increased its finances by several hundred dollars; that it has increased its membership by fifty-four new men, thus bringing much new blood, new vigor, and new life. I am proud that throughout this time our Board of Trustees has held such a censorship that we can well feel each of these men will not only be a help in many ways, but without exception an honor to our Association.

I am gratified that this work should have fallen upon the shoulders of one whom I feel full well is deserving of the honor, and it is undoubtedly an honor, as herein much harm may be done or much good accomplished. In fact, the destiny of your Association lies in the hands of your Secretaries. I transfer with pleasure the duties and emoluments of the office to

Dr. Ranck, knowing that through his efforts, backed by his energy and perseverance, our finances, membership, and patronage will increase.

I have the following to ask you, gentlemen: First, that you will all read your book of by-laws, especially that part relating to dues, then those of you who have not already done so turn over a new leaf and pay your dues in advance; secondly, that you will extend to Dr. Ranck, as you have to me, every courtesy and help.

I will give you a brief *résumé* of my work the past three years. In March, 1897, the State of Pennsylvania had twenty-six counties represented in our State Association; March of 1900 there are thirty-nine counties represented in our Association. In March, 1897, we had 117 names on our active membership list; we now have 132 *good*, active members (with the emphasis on the good) on our active membership-list; also 33 names which I have submitted to the Board of Censors for expulsion, thus purging our list of all delinquents; also a list of three who have left our State while in good standing, and whom I suggest be listed as corresponding or associate members. Our honorary membership-list remains the same, as honors in our Association come hard. Our list of deceased members is increased by the death of Dr. John J. Smith, of Chambersburg. Our list of resignations has increased by three, viz., W. A. Heckenberger, of Catasauqua; E. Sturge, of Scranton, and W. L. Zuill, of Philadelphia. Our list of expelled members will, I fear, be at this time augmented by thirty-three new ones.

| | | |
|---|---|---|
| I have received as dues since March of 1897 | $509.00 | |
| I have received as membership fee from 54 new members at $5 each | 270.00 | |
| Making a total of | | $779.00 |
| My expenses have been as follows: | | |
| For year ending March, 1898 | $103.05 | |
| For year ending March 7, 1899 | 185.05 | |
| (This amount includes the printing and distribution of the By-laws.) | | |
| For year ending March 7, 1900 | 94.00 | |
| (The check for balance of $86.25, which I received from my predecessor, Dr. F. S. Allen, I enclosed to our Treasurer, Dr. Francis Bridge.) | | |
| I hold receipts for other moneys paid by me to Dr. Bridge to the amount of | 317.50 | |
| | | 699.60 |
| Leaving a balance in my hands due the Association of | | $79.40 |
| Moneys received by check from F. S. Allen | $86.25 | |
| Moneys received as dues | 509.00 | |
| Moneys received as membership fees | 270.00 | |
| | | 865.25 |
| Paid out check of F. S. Allen | $86.25 | |
| Moneys paid Dr. Bridge from dues— | | |
| Membership fees, as follows: March 7, 1899 | $100.00 | |
| March 18, 1899 | 42.50 | |
| March 6, 1900 | 175.00 | |
| Expenses | 382.10 | |
| Balance in my hands | 79.40 | |
| | | $865.25 |

Respectfully submitted, March 7, 1900.

W. L. RHOADS,
Corresponding Secretary

Dr. Charles Williams then read an interesting paper on the subject of "Antitoxin Treatment for Distemper in Horses."

The Board of Trustees then made their annual report.

The Committee on Resolutions reported the following :

*Reappointment of State Veterinarian.*

WHEREAS, The Hon. William A. Stone, Governor of our State, has since our last meeting appointed our esteemed colleague, Dr. Leonard Pearson, to the position of State Veterinarian; and, WHEREAS, In so doing he has not only observed the wishes of the representatives of the live-stock interests in our State, but has also met the desire and request of this organization; therefore be it *Resolved*, That we assure the Governor of our sincere appreciation of this action, and feel assured that the administration of the State Veterinarian will prove the best evidence of the wisdom of his selection.

*State Board of Veterinary Examiners.*

WHEREAS, The Governor, Hon. William A. Stone, has recognized the valuable services of our colleague, Dr. J. W. Sallade, and has reappointed him on the State Veterinary Medical Examining Board, and has, moreovers appointed the nominees of this Association, Drs. W. H. Ridge and Jacob Helmer, to fill the remaining vacancies on the Examining Board, and by this means has advanced the interests of the veterinary profession; be it *Resolved*, That the thanks of this Association are hereby tendered His Excellency, Governor Stone, who has acted in this matter for the best interest, of the public and entirely in accord with the wishes of this Association.

*Good Roads.*

WHEREAS, The matter of good roads in Pennsylvania is a subject of such extreme importance that it constitutes one of the pressing public questions of the day, and the value of good roads is no longer a matter of conjecture or one upon which there is justifiable difference of opinion; and WHEREAS, The efficiency and value of horses and the comfort and pleasure of using them are augmented by good roads; be it *Resolved*, That we unqualifiedly approve the efforts being made by the State Department of Agriculture in behalf of good roads, and earnestly commend the excellent work already accomplished in this direction by the Secretary of Agriculture, Professor John Hamilton.

*Veterinary Department's Facilities.*

WHEREAS, Our Association has again been favored by the University of Pennsylvania's Veterinary Department with its splendid facilities for the holding of our annual meeting; and WHEREAS, we desire to express our grateful appreciation of these privileges afforded us, the courtesy and kindness of the employés of the department, and hereby tender our sincere thanks for the same.

*Our Post-Graduate Course.*

WHEREAS, The initial effort to establish a post-graduate course of instruction in connection with the work of our State Live-stock Sanitary Board at the University of Pennsylvania Veterinary Department; and WHEREAS, The success attending this effort has so far exceeded in every direction the most sanguine anticipations and hopes of those directing the work; be it *Resolved*, That we tender our University, her corps of instructors, our State Veterinarian, who has so successfully directed the same, and State Association our cordial indorsement of the same, our congratulations on the success of the effort and our unanimous request that this course become an annual one.

The new officers were then duly installed with the exception of President Harger, whose place was filled by second Vice-President Dr. L. O. Lusson.

The Association then adjourned to visit the H. K. MulfordLaboratories, which closed the annual session for 1900.

